# The oribatid mite genus *Macrogena* (Acari, Oribatida, Ceratozetidae), with description of two new species from New Zealand

**DOI:** 10.3897/zookeys.506.9796

**Published:** 2015-05-28

**Authors:** Sergey G. Ermilov, Maria A. Minor

**Affiliations:** 1 Tyumen State University, Tyumen, Russia; 2 Institute of Agriculture & Environment, Massey University, Palmerston North, New Zealand

**Keywords:** Oribatid mites, *Macrogena*, new species, generic diagnosis, key, New Zealand

## Abstract

Two new species of oribatid mites of the genus *Macrogena* (Oribatida, Ceratozetidae) are described from alpine soils of the South Island of New Zealand. *Macrogena brevisensilla* **sp. n.** and *Macrogena abbreviata* **sp. n.** differ from all species of this genus by the tridactylous legs and by the comparatively short interlamellar setae, respectively. New generic diagnosis and an identification key to the known species of *Macrogena* are provided.

## Introduction

*Macrogena* is an oribatid mite genus of the family Ceratozetidae (Acari, Oribatida) which was proposed by Wallwork (1966) with *Macrogena monodactyla* Wallwork, 1966 as type species. At present, three species are known^1^: *Macrogena crassa* Hammer, 1967, *Macrogena rudentiger* Hammer, 1967 (both from New Zealand) and *Macrogena monodactyla* Wallwork, 1966 (from the Antarctic region).

During the recent study of oribatid mite fauna of the high alpine zone of several mountain ranges in New Zealand (Central Otago, South Island), we discovered two new species of the genus *Macrogena*; both species were common and abundant in the collected material. Additionally, we propose a new generic diagnosis for *Macrogena*, and provide an identification key for all known species of this genus.

## Materials and methods

The collection locality and habitat for each new species are given in the respective “*Material examined*” sections.

Specimens were mounted in lactic acid on temporary cavity slides for measurement and illustration. The body length was measured in lateral view, from the tip of the rostrum to the posterior edge of the ventral plate. Notogastral width refers to the maximum width in dorsal aspect. Lengths of body setae were measured in lateral aspect. All body measurements are presented in micrometers. Formulas for leg setation are given in parentheses according to the sequence trochanter–femur–genu–tibia–tarsus (famulus included). Formulas for leg solenidia are given in square brackets according to the sequence genu–tibia–tarsus. General terminology used in this paper follows that of Grandjean (summarized by Norton and Behan-Pelletier 2009). Drawings were made with a drawing tube using a Carl Zeiss transmission light microscope “Axioskop-2 Plus”. Images were obtained with an AxioCam ICc3 camera using a Carl Zeiss transmission light microscope “Axio Lab.A1”.

## Taxonomy

### Genus Macrogena

Taxon classificationAnimaliaOribatidaCeratozetidae

Wallwork, 1966

#### Type species.

*Macrogena monodactyla* Wallwork, 1966

#### Diagnosis

(partially based on data from Wallwork 1966; Hammer 1967). Ceratozetidae with rostrum with medial rectangular ledge formed by two lateral incisions; rostral setae inserted dorsally or dorso-laterally on prodorsum; lamellar and interlamellar setae strong, straight; bothridial setae fusiform or globular; lamellae large, with short cusps, connected by translamella; tutoria and genal teeth long, reach the level of insertions of rostral setae; dorsophragmata fused medially; notogaster with three or four pairs of porose areas; ten pairs of short and thin notogastral setae; five pairs of genital, one pair of aggenital, two pair of anal, and three pairs of adanal setae; legs mono- or tridactylous.

### Macrogena brevisensilla sp. n.

Taxon classificationAnimaliaOribatidaCeratozetidae

http://zoobank.org/7D7732BB-EF8C-47F5-8A14-23903114CE77

Figs 1–4 , 5–9 , 10–13 , 14–22

#### Diagnosis.

Body size: 315–332 × 182–199. Lamellar cusps without teeth. Translamella broad. Rostral setae dilated in medio-distal parts, ciliated. Lamellar and interlamellar setae long, thickened, densely barbed. Bothridial setae globular. Tutoria broadly triangular. Four pairs of notogastral porose areas present. Notogastral setae short, thin. Epimeral setae *1c* thickened, barbed. Tridactylous.

#### Description.

*Measurements*. Body length: 332 (holotype: female), 315–332 (six paratypes: three females, three males); notogaster width: 182 (holotype), 182–199 (six paratypes).

*Integument*. Body color light brown to brown. Body surface punctate (visible under high magnification). Lamellae, epimeral region, pedotecta I and subcapitular mentum with striae.

*Prodorsum*. Anterior edge of medial ledge of rostrum slightly wavy, lateral incisions very narrow. Lamellae shorter than half of prodorsum. Lamellar cusps without teeth. Translamella straight, broad. Rostral setae (*ro*, 32–41) dilated in medio-distal parts, ciliated. Lamellar (*le*, 49–57) and interlamellar (*in*, 82–90) setae thickened, densely barbed. Lamellar setae sometimes slightly dilated medio-distally. Bothridial setae (*ss*, 22–26) globular, with short stalk (4–6) and longer, indistinctly barbed head (18–20). Tutoria (*tu*) broadly triangular distally. Exobothridial setae (*ex*, 4) thin, smooth.

*Notogaster*. Anterior margin convex medially. Pteromorphs broadly rounded laterally. Porose areas *Am* elongate oval. Dorsophragmata (*D*) of medium size. Four pairs of porose areas present, all rounded: *Aa* (8), *A1*, *A2* and *A3* (6). Notogastral setae thin, smooth, *c* (12) little longer than other nine pairs (6–8). Lyrifissures *ia*, *im*, *ip*, *ih* and *ips* distinct. Opisthonotal gland openings (*gla*) located posteriorly to *im*.

*Gnathosoma*. Subcapitulum longer than wide (86 × 61–65). Subcapitular setae *h* (4–6) thin, smooth; *a* (12–16) and *m* (18–20) setiform, slightly barbed. Adoral setae (*or*_1_, *or*_2_, 8–10) simple, densely barbed. Palps (53–61) with setation 0–2–1–3–9(+ω). Solenidion attached to eupathidium, both located on dorsal tubercle. Chelicerae (90–94) with two simple, barbed setae; *cha* (28–32) longer than *chb* (16–20). Trägårdh’s organ (Tg) long, tapered.

*Epimeral and lateral podosomal regions*. Pedotecta I (Pd I) large, concave in dorsal view. Pedotecta II (Pd II) of medium size, triangular, rounded distally in ventral view. All pedotecta scale-like in lateral view. Genal teeth (*gt*) elongate narrowly triangular. Apodemes 1, 2, sejugal and 3 distinctly developed. Epimeral setal formula 3–1–2–2. Epimeral setae *1c* (10) thickened, barbed; other setae (4–6) thin, smooth. Custodia (*cus*) with long, pointed tips. Discidia (*dis*) triangular. Circumpedal carinae (*cp*) distinct.

*Anogenital region*. Genital (*g*_1_–*g*_5_, 4–6), aggenital (*ag*, 4–6), anal (*an*_1_, *an*_2_, 4–6) and adanal (*ad*_1_–*ad*_3_, 6–8) setae thin, smooth. Lyrifissures *iad* located close to anal aperture, in paraanal position. Ovipositor elongated (102–110 × 28), blades (45–49) shorter than length of distal section (beyond middle fold; 57–61). Each of three blades with four straight, smooth setae, ψ_1_ ≈ τ_1_ (32) longer than ψ_2_ ≈ τ_a_ ≈ τ_b_ ≈ τ_c_ (14–16). Six coronal simple setae (*k*, 8) present.

*Legs*. Tridactylous. Medial claw thicker than two laterals; all indistinctly serrate dorsally. Genua I and II, and femora II with antero-ventral tooth (*t*). Formulae of leg setation and solenidia: I (1–5–3–3–18) [1–2–2], II (1–5–3–4–15) [1–1–2], III (2–2–1–3–15) [1–1–0], IV (1–2–2–3–12) [0–1–0]; homology of setae and solenidia as indicated in Table 1. Famulus (ε) short, blunted. Setae *l*’’ on tibiae and genua I, II thick.

**Table 1.** Leg setation and solenidia of adult *Macrogena brevisensilla* sp. n.

Leg	Trochanter	Femur	Genu	Tibia	Tarsus
I	*v*’	*d*, *(l)*, *bv*’’, *v*’’	*(l)*, *v*’, σ	*(l)*, *v*’, φ_1_, φ_2_	*(ft)*, *(tc)*, *(it)*, *(p)*, *(u)*, *(a)*, *s*, *(pv)*, *(pl)*, ε, ω_1_, ω_2_
II	*v*’	*d*, *(l)*, *bv*’’, *v*’’	*(l)*, *v*’, σ	*(l)*, *(v)*, φ	*(ft)*, *(tc)*, *(it)*, *(p)*, *(u)*, *(a)*, *s*, *(pv)*, ω_1_, ω_2_
III	*l*’, *v*’	*d*, *ev*’	*l*’, σ	*l*’, *(v)*, φ	*(ft)*, *(tc)*, *(it)*, *(p)*, *(u)*, *(a)*, *s*, *(pv)*
IV	*v*’	*d*, *ev*’	*d*, *l*’	*l*’, *(v)*, φ	*ft*’’, *(tc)*, *(p)*, *(u)*, *(a)*, *s*, *(pv)*

Note: Roman letters refer to normal setae, Greek letters to solenidia (except for ε = famulus). Single prime (‘) marks setae on the anterior and double prime (“) setae on the posterior side of a given leg segment. Parentheses refer to a pair of setae.

#### Material examined.

Holotype (female) and six paratypes (three females and three males): New Zealand, South Island, Central Otago, The Remarkables, 45°3'38"S, 168°48'43"E, 1867 m a.s.l., in the soil and debris under *Raoulia* sp. cushion, 19 February 2014, collected by M. Minor.

#### Type deposition.

The holotype and two paratypes are deposited in the New Zealand National Arthropod Collection, Auckland, New Zealand; two paratypes are deposited in the collection of the Senckenberg Institution, Frankfurt, Germany; two paratypes are deposited in the collection of the Tyumen State University Museum of Zoology, Tyumen, Russia.

#### Etymology.

The specific name *brevisensilla* refers to the short bothridial setae (sensilla).

#### Remarks.

*Macrogena brevisensilla* sp. n. differs from all species of this genus by the tridactylous legs.

Wallwork (1966) considered monodactylous legs as the generic character of *Macrogena*. The new species has tridactylous legs, however, all other morphological traits correspond to the other species of this genus. Thus, we included *Macrogena brevisensilla* sp. n. in *Macrogena*, and included alternatively tridactylous legs in the revised generic diagnosis.

**Figures 1–4.**
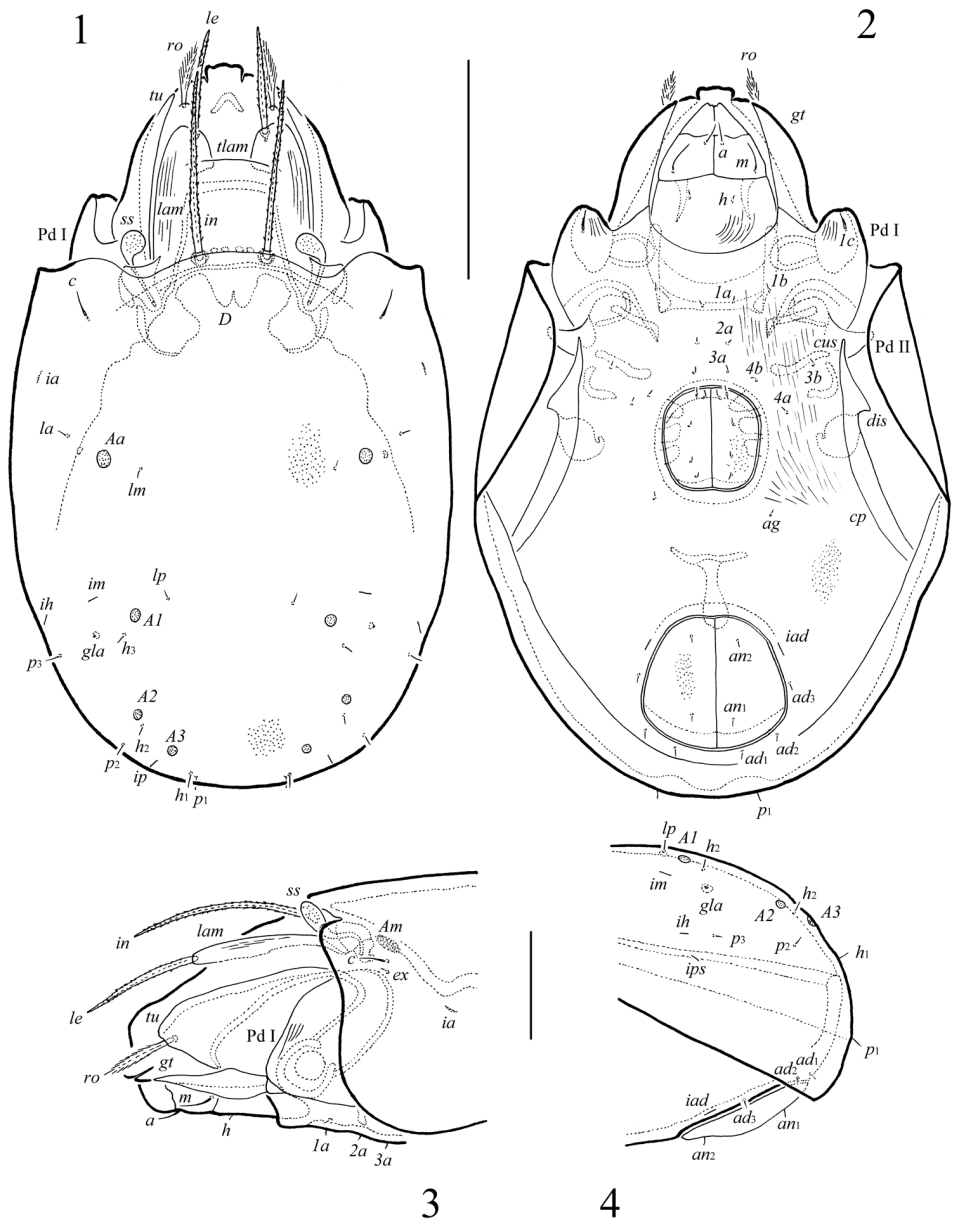
*Macrogena brevisensilla* sp. n., adult: **1** dorsal view **2** ventral view (legs not shown) **3** lateral view of anterior part of body (leg I not shown) **4** lateral view of posterior part of body. Scale bars 100 µm (**1, 2**), 50 µm (**3, 4**).

**Figures 5–9.**
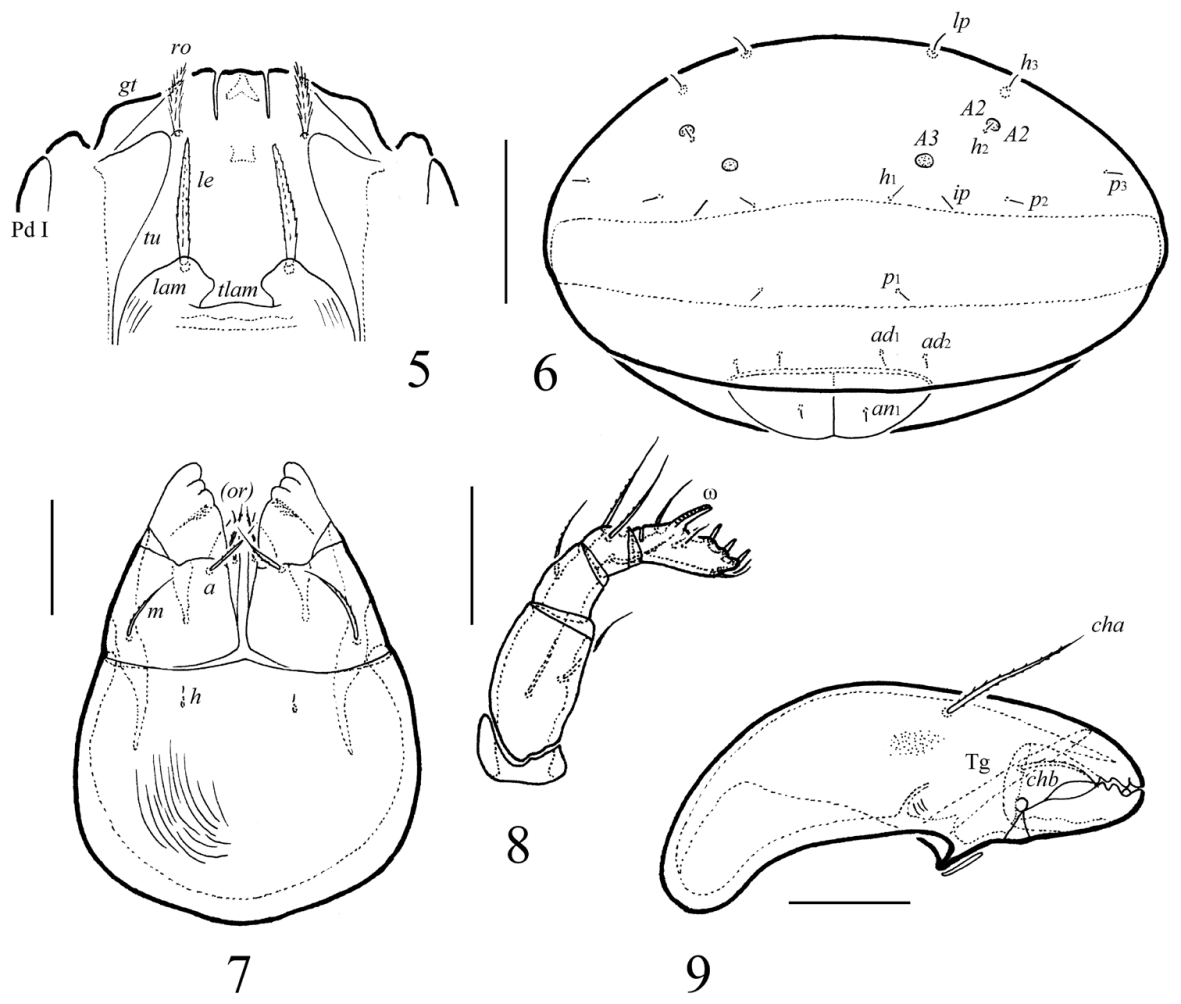
*Macrogena brevisensilla* sp. n., adult: **5** frontal view of prodorsum **6** posterior view **7** subcapitulum, ventral view **8** palp **9** chelicera. Scale bars 50 µm (**5, 6**), 20 µm (**7–9**).

**Figures 10–13.**
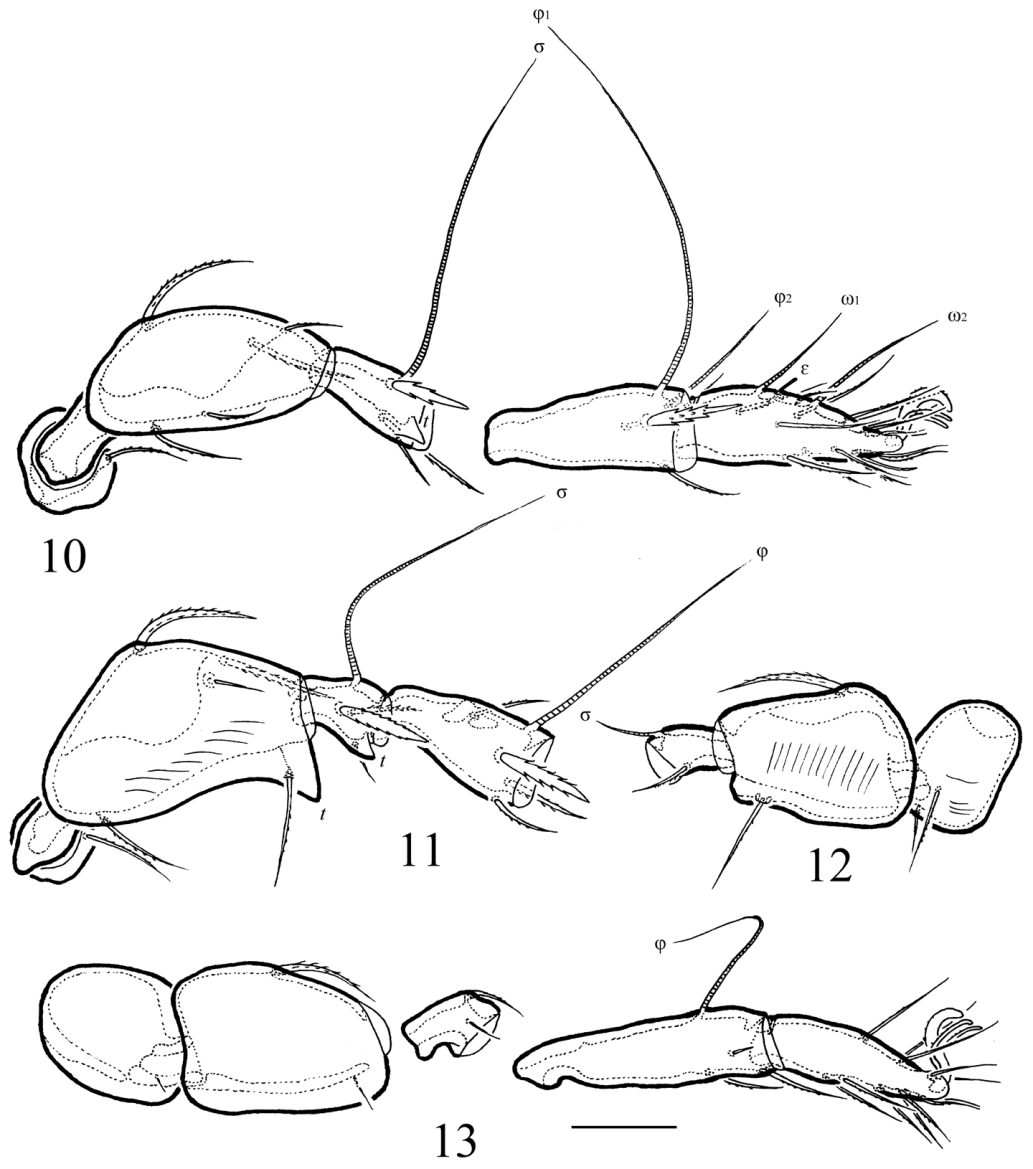
*Macrogena brevisensilla* sp. n., adult: **10** leg I, right, antiaxial view **11** tibia, genu, femur and trochanter of leg II, right, antiaxial view **12** genu, femur and trochanter of leg III, right, antiaxial view **13** leg IV, left, antiaxial view. Scale bar 20 µm.

**Figures 14–22.**
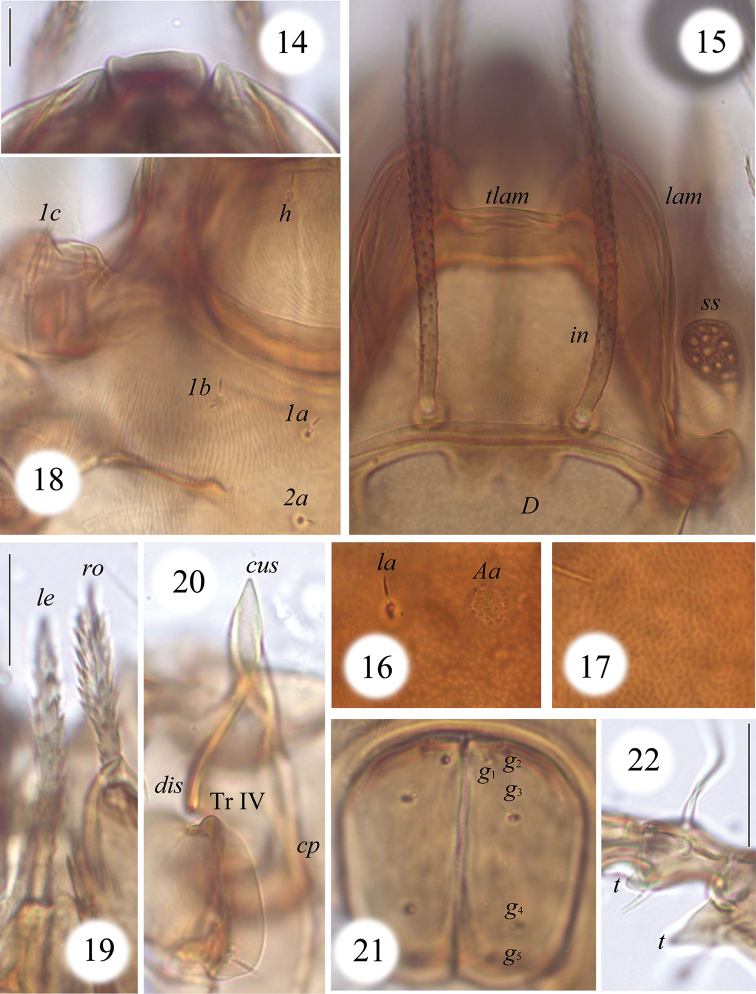
*Macrogena brevisensilla* sp. n., dissected adult, microscope images: **14** rostrum, dorsal view **15** medio-basal part of prodorsum and medio-anterior part of notogaster **16** notogastral porose area *Aa* and seta *la* **17** microporose in medial part of notogaster **18** right parts of subcapitular mentum and epimere I **19** rostral and lamellar setae **20** custodium and discidium **21** genital plates **22** ventral teeth on leg II (left, antiaxial view). Scale bar 20 µm.

### Macrogena abbreviata sp. n.

Taxon classificationAnimaliaOribatidaCeratozetidae

http://zoobank.org/2F9CD29C-B10C-42A2-B3F5-5C13EF4954BC

Figs 23–25 , 26–30 , 31–34 , 35–44

#### Diagnosis.

Body size: 254–291 × 143–151. Lamellar cusps without teeth. Translamella of medium thickness. Rostral setae setiform, ciliated. Lamellar and interlamellar setae of medium size, thickened, barbed. Bothridial setae fusiform. Three pairs of notogastral porose areas present. Notogastral setae short, thin. Monodactylous.

#### Description.

*Measurements*. Body length: 270 (holotype: female), 254–291 (six paratypes: three females, three males); notogaster width: 143 (holotype), 143–151 (six paratypes).

*Integument*. Body color light brown to brown. Body surface punctate (visible under high magnification). Lamellae and pedotecta I striate; epimeral region also with longitudinal striae, however it is visible only in dissected specimens.

*Prodorsum*. Medial ledge of rostrum truncated, lateral incisions well visible. Lamellae shorter than half of prodorsum. Lamellar cusps without teeth. Translamella straight, thickened. Rostral setae (24–28) setiform, ciliated. Lamellar (18–20) and interlamellar (32–36) setae thickened, usually indistinctly barbed (rarely with sparse, strong barbs). Bothridial setae (22–26) fusiform, with short stalk (6) and longer, indistinctly barbed head (16–20). Tutoria narrowly triangular distally, slightly rounded, sometimes pointed. Exobothridial setae (4) thin, smooth.

*Notogaster*. Anterior margin convex medially. Pteromorphs broadly rounded laterally. Porose areas *Am* elongate oval. Dorsophragmata of medium size. Three pairs of porose areas present, all rounded: *Aa* (6–8), *A2* and *A3* (4–6). Notogastral setae (12–14) thin, smooth. Lyrifissures distinct. Opisthonotal gland openings located posteriorly to *im*.

*Gnathosoma*. Subcapitulum longer than wide (65–69 × 45–53). Subcapitular setae thin, slightly barbed; *m* (18–20) longer than *a* (12–16) and *h* (8–10). Adoral setae (8) simple, densely barbed. Palps (45) with setation 0–2–1–3–9(+ω). Solenidion attached to eupathidium, both located on dorsal tubercle. Chelicerae (73–77) with two simple, barbed setae; *cha* (28) longer than *chb* (14–16). Trägårdh’s organ long, tapered.

*Epimeral and lateral podosomal regions*. Pedotecta I large, concave in dorsal view. Pedotecta II of medium size, triangular, rounded distally in ventral view. All pedotecta scale-like in lateral view. Genal teeth elongate narrowly triangular. Apodemes 1, 2, sejugal and 3 distinctly developed. Epimeral setal formula 3–1–2–2. Epimeral setae (8) thin, smooth. Custodia with long, pointed tips. Discidia triangular. Circumpedal carinae distinct.

*Anogenital region*. Genital (8–12), aggenital (8–12), anal (8–12) and adanal (12–14) setae thin, smooth. Lyrifissures *iad* located close to anal aperture, in inverse apoanal position. Ovipositor elongated (106–110 × 28), blades (45–49) shorter than length of distal section (beyond middle fold; 57–61). Each of three blades with four straight, smooth setae, ψ_1_ ≈ τ_1_ (24–28) longer than ψ_2_ ≈ τ_a_ ≈ τ_b_ ≈ τ_c_ (12–16). Six coronal simple setae (8) present.

*Legs*. Monodactylous. Claws indistinctly serrate dorsally. Femora II with antero-ventral tooth (*t*). Formulae of leg setation and solenidia: I (1–4–3–4–18) [1–2–2], II (1–5–3–4–15) [1–1–2], III (2–2–1–3–15) [1–1–0], IV (1–2–2–2–12) [0–1–0]; homology of setae and solenidia as indicated in Table 2. Famulus (ε) short, blunted. Setae *l*’’ on genua I, II thick.

**Table 2.** Leg setation and solenidia of adult *Macrogena abbreviata* sp. n.

Leg	Trochanter	Femur	Genu	Tibia	Tarsus
I	*v*’	*d*, *(l)*, *bv*’’	*(l)*, *v*’, σ	*(l)*, *(v)*, φ_1_, φ_2_	*(ft)*, *(tc)*, *(it)*, *(p)*, *(u)*, *(a)*, *s*, *(pv)*, *(pl)*, ε, ω_1_, ω_2_
II	*v*’	*d*, *(l)*, *bv*’’, *v*’’	*(l)*, *v*’, σ	*(l)*, *(v)*, φ	*(ft)*, *(tc)*, *(it)*, *(p)*, *(u)*, *(a)*, *s*, *(pv)*, ω_1_, ω_2_
III	*l*’, *v*’	*d*, *ev*’	*l*’, σ	*l*’, *(v)*, φ	*(ft)*, *(tc)*, *(it)*, *(p)*, *(u)*, *(a)*, *s*, *(pv)*
IV	*v*’	*d*, *ev*’	*d*, *l*’	*(v)*, φ	*ft*’’, *(tc)*, *(p)*, *(u)*, *(a)*, *s*, *(pv)*

Note: See Table 1 for explanations.

#### Material examined.

Holotype (female) and six paratypes (three females and three males): New Zealand, South Island, Central Otago, Old Man’s Range, 45°19'24"S, 169°12'28"E, 1655 m a.s.l., in the bare soil with some lichen outside of *Dracophyllum muscoides* cushion, 17 February 2014, collected by M. Minor.

#### Type deposition.

The holotype and two paratypes are deposited in the New Zealand National Arthropod Collection, Auckland, New Zealand; two paratypes are deposited in the collection of the Senckenberg Institution, Frankfurt, Germany; two paratypes are deposited in the collection of the Tyumen State University Museum of Zoology, Tyumen, Russia.

#### Etymology.

The specific name *abbreviata* refers to the comparatively short interlamellar setae of this species.

#### Remarks.

*Macrogena abbreviata* sp. n. differs from all other species of this genus by the short interlamellar setae, which do not reach the lamellar cusps.

**Figures 23–25.**
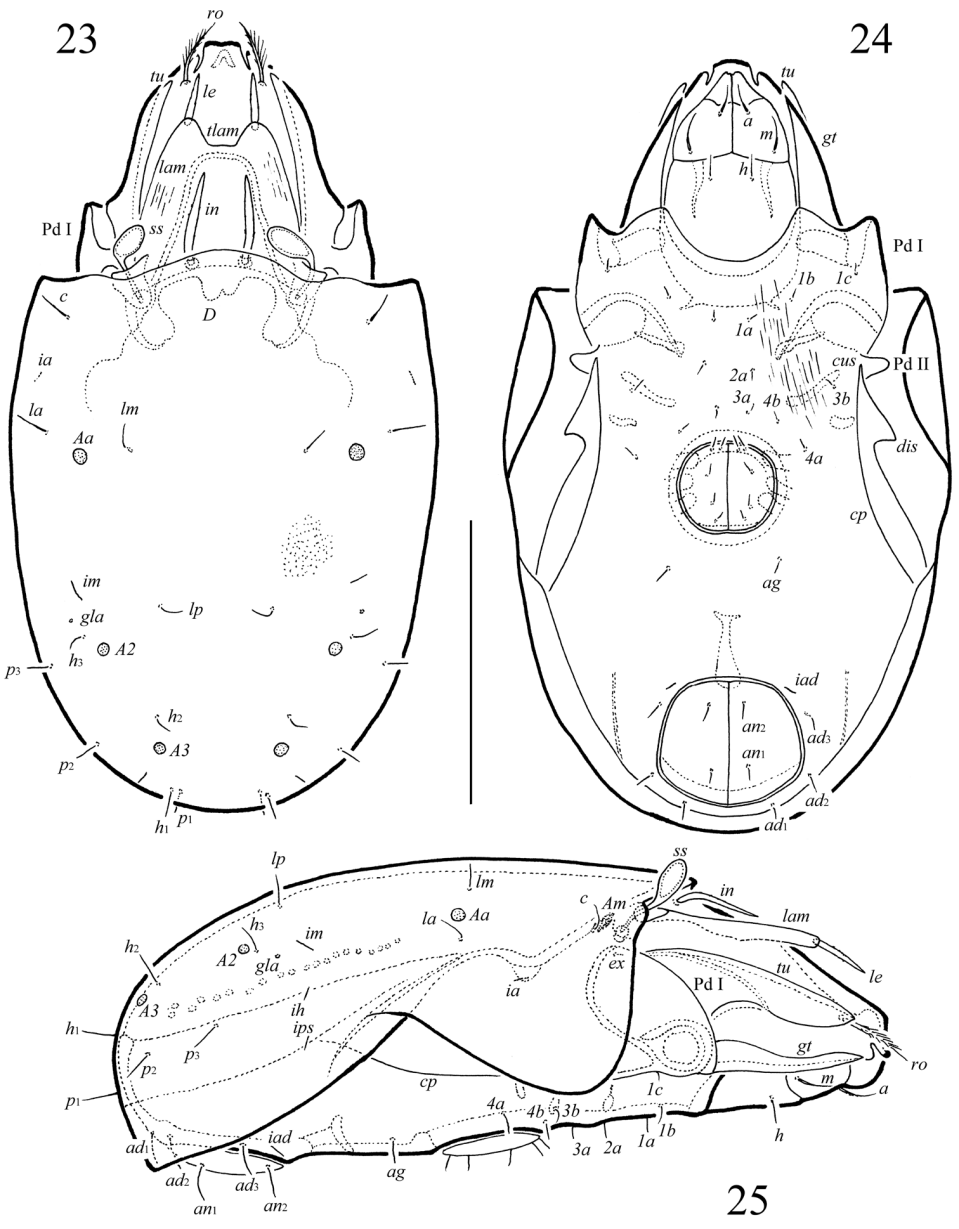
*Macrogena abbreviata* sp. n., adult: **23** dorsal view **24** ventral view (legs not shown) **25** lateral view (leg I not shown). Scale bar 100 µm.

**Figures 26–30.**
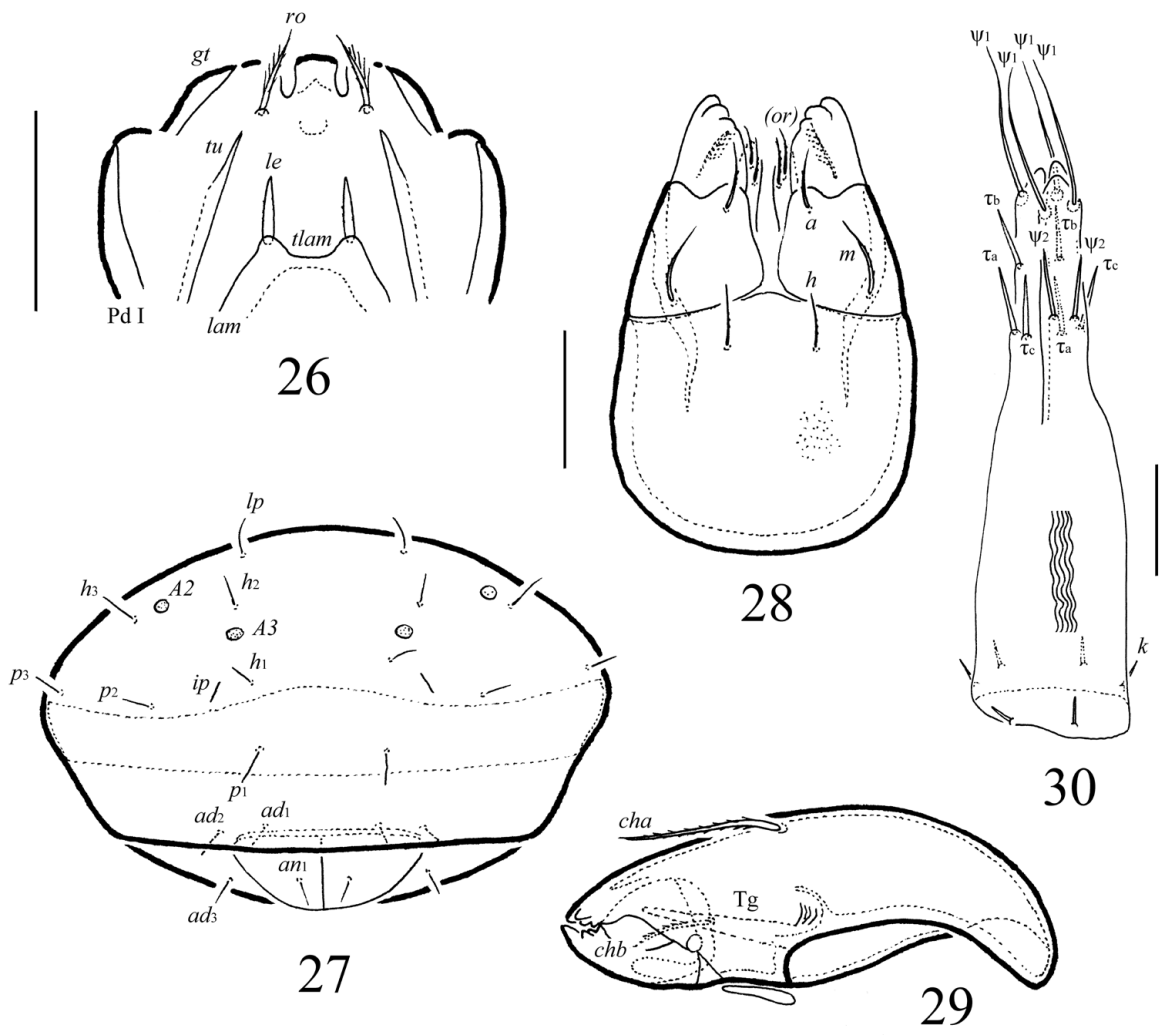
*Macrogena abbreviata* sp. n., adult: **26** frontal view of prodorsum **27** posterior view **28** subcapitulum, ventral view **29** chelicera **30** ovipositor. Scale bars 50 µm (**26, 27**), 20 µm (**28–30**).

**Figures 31–34.**
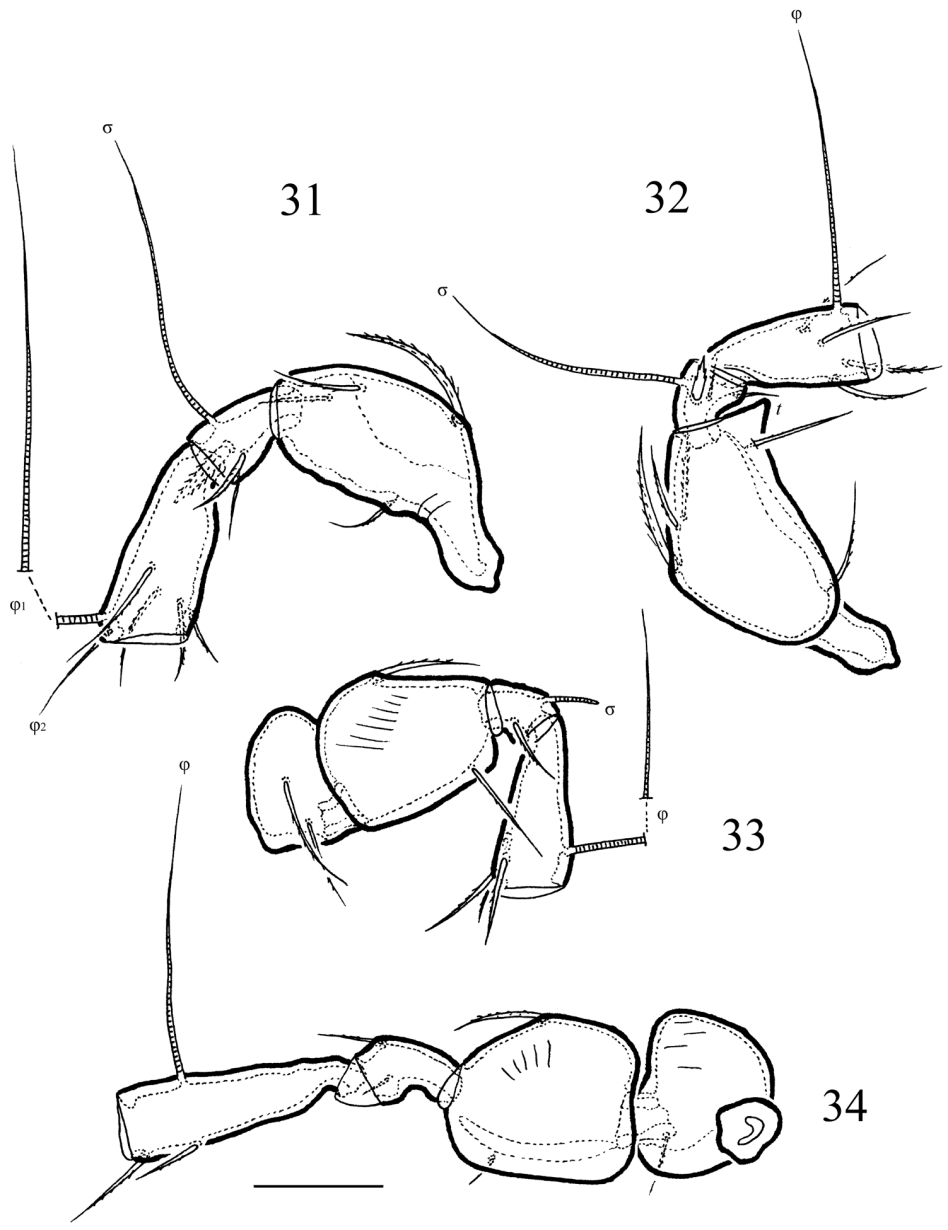
*Macrogena abbreviata* sp. n., adult: **31** tibia, genu and femur of leg I, right, paraxial view **32** tibia, genu and femur of leg II, right, antiaxial view **33** tibia, genu, femur and trochanter of leg III, left, antiaxial view **34** tibia, genu, femur and trochanter of leg IV, left, paraxial view. Scale bar 20 µm.

**Figures 35–44.**
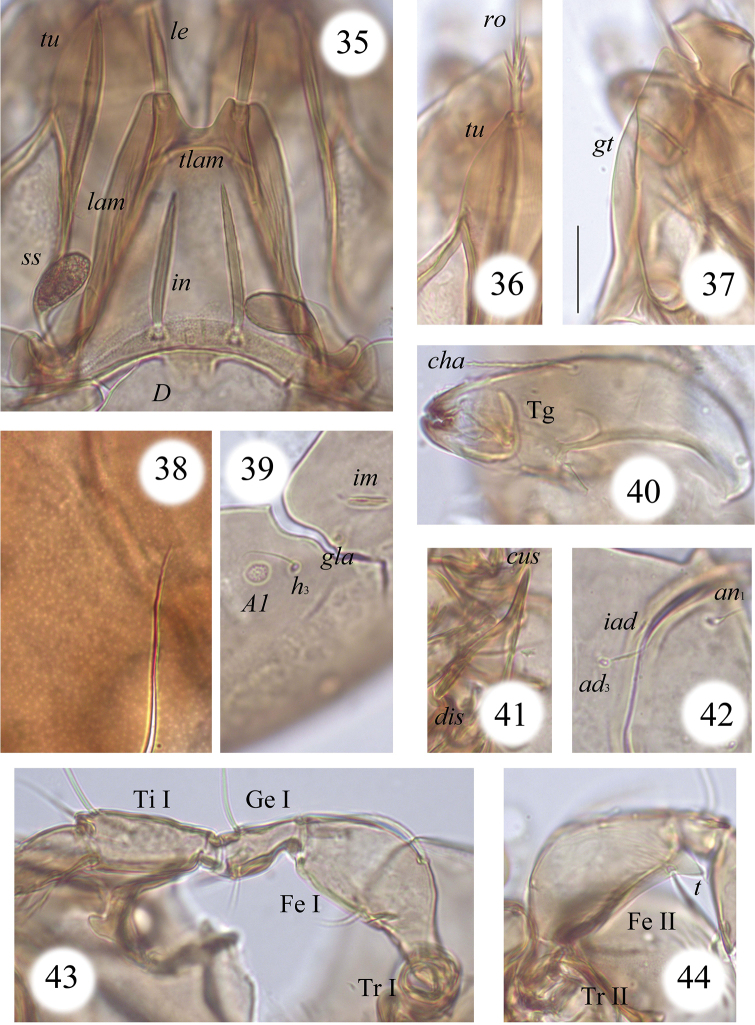
*Macrogena abbreviata* sp. n., dissected adult, microscope images: **35** medio-basal part of prodorsum and medio-anterior part of notogaster **36** rostral seta and medio-anterior part of tutorium **37** genal tooth and rostral incision **38** microporose in medial part of notogaster **39** notogastral porose area *Aa*, seta *h*_3_, lyrifissure *im* and opisthonotal gland opening **40** chelicera **41** custodium and discidium **42** anal seta *an*_2_, adanal seta *ad*_3_ and lyrifissure *iad* **43** leg I (except anterior part of tarsus), right, paraxial view **44** ventral tooth on leg II (right, antiaxial view). Scale bar 20 µm.

### Key to known species *Macrogena*

**Table:** 

1	Legs tridactylous; four pairs of porose areas present; body size: 315–332 × 182–199	***Macrogena brevisensilla* sp. n.** Distribution: New Zealand.
–	Legs monodactylous; three pairs of porose areas present	**2**
2	Bothridial setae with a short stalk (half or less than the length of head)	**3**
–	Bothridial setae with a long stalk (similar or longer than the length of head)	**4**
3	Lamellar cusps without teeth; interlamellar setae of medium size, not reaching the lamellar cusps; body size: 254–291 × 143–151	***Macrogena abbreviata* sp. n.** Distribution: New Zealand.
–	Lamellar cusps with lateral teeth; interlamellar setae long, reaching the lamellar cusps; body size: 308–330 × 198–213	***Macrogena monodactyla* Wallwork, 1966.** Distribution: Antarctic region.
4	Lamellar cusps with lateral teeth; interlamellar setae not reaching the rostrum; body length: 240	***Macrogena rudentiger* Hammer, 1967.** Distribution: New Zealand.
–	Lamellar cusps without teeth; interlamellar setae reaching the rostrum; body length: 280	***Macrogena crassa* Hammer, 1967.** Distribution: New Zealand.

## Supplementary Material

XML Treatment for Macrogena

XML Treatment for Macrogena brevisensilla

XML Treatment for Macrogena abbreviata

## Footnotes

Subías (2004, updated 2015) included in *Macrogena* the following three species: *Mycobates minor* Subías, Kahwash & Ruiz, 1990 from the Mediterranean, *Lophozetes truncatus* Balogh, 1985 from Australia and *Safrobates miniporus* Mahunka, 1989 from Tasmania. However, *Mycobates minor* has rostral setae inserted laterally on prodorsum, dorsophragmata separated medially and six pairs of genital setae; *Lophozetes truncatus* and *Safrobates miniporus* have lamellar cusps fused medially. Hence, the morphological characters of these species do not correspond absolutely to the generic diagnosis of *Macrogena*, therefore their systematic position need further investigations.
